# P02.143. Life, liberty, and the pursuit of mindfulness in higher education settings

**DOI:** 10.1186/1472-6882-12-S1-P199

**Published:** 2012-06-12

**Authors:** L Teitelbaum, J Ouellette

**Affiliations:** 1 SUNY Cortland, Fayetteville, USA

## Purpose

The pursuit of wellness can be a challenge for many college students who are under constant pressures to succeed academically as well as socially. Low levels of psychological wellness can compromise students’ concentration and academic performance and, in extreme cases, can lead them to contemplate suicide, making wellness initiatives a critical consideration. Mindfulness meditation interventions used in higher education settings have been found to reduce stress, anxiety, and depression. This pilot study was undertaken to assess mindfulness and other key factors that could be targeted to improve psychological wellness on campus.

## Methods

Undergraduate students (n=49) enrolled in an introductory psychology class attending a State University in NY completed measures of Mindfulness (MAAS; Brown & Ryan, 2003); Perceived Stress (Cohen & Williamson, 1988 ); Self-Esteem (Rosenberg; 1989); Psychological Symptoms (SA-45; Strategic Advantage, 1998); and Quality of Sleep (modified Pittsburgh Sleep Inventory). Informed consent for each student was obtained.

## Results

Pearson correlation analyses revealed that psychological symptoms were negatively correlated with mindfulness (r = -.33; p<.05) and self-esteem (r= -.41; p<.01), and positively correlated with perceived stress (r = .29; p<.05). In addition, poor sleep patterns significantly contributed to higher levels of perceived stress (r = -.54. p<.01).

## Conclusion

This pilot study revealed that (a) increased mindfulness; (b) lower levels of stress; and (c) higher self-esteem were all related to psychological wellbeing. These findings add to a burgeoning body of literature that suggests higher levels of mindfulness are related to greater emotional wellbeing. Mindfulness can be enhanced through training and practice and has been found to decrease stress and improve sleep quality. Thus, these results support the implementation of mindfulness-based meditation interventions that can decrease stress, as well as increase mindfulness, self-esteem, quality of sleep and psychological wellbeing in this at risk population.

